# Pharmacological Approaches to the Treatment of Dementia in Down Syndrome: A Systematic Review of Randomized Clinical Studies

**DOI:** 10.3390/molecules27103244

**Published:** 2022-05-19

**Authors:** Laura Cavalcanti de Oliveira, Daniele de Paula Faria

**Affiliations:** Laboratory of Nuclear Medicine (LIM43), Department of Radiology and Oncology, Faculdade de Medicina FMUSP, Universidade de Sao Paulo, Sao Paulo 05403-911, SP, Brazil; lauracavalcanti459@gmail.com

**Keywords:** down syndrome, trisomy 21, Alzheimer’s disease, dementia, pharmacological treatment, cognitive decline

## Abstract

Down Syndrome (DS) is considered the most frequent form of Intellectual Disability, with important expressions of cognitive decline and early dementia. Studies on potential treatments for dementia in this population are still scarce. Thus, the current review aims to synthesize the different pharmacological approaches that already exist in the literature, which focus on improving the set of symptoms related to dementia in people with DS. A total of six studies were included, evaluating the application of supplemental antioxidant therapies, such as alpha-tocopherol; the use of acetylcholinesterase inhibitor drugs, such as donepezil; N-methyl-d-aspartate (NMDA) receptor antagonists, such as memantine; and the use of vitamin E and a fast-acting intranasal insulin. Two studies observed important positive changes related to some general functions in people with DS (referring to donepezil). In the majority of studies, the use of pharmacological therapies did not lead to improvement in the set of symptoms related to dementia, such as memory and general functionality, in the population with DS.

## 1. Introduction

Down syndrome (DS) is considered the most frequent form of intellectual disability, with important expressions of cognitive decline and early dementia [1,2]. There has been an increase in the life expectancy of this population in recent years (to around 55 years), mainly influenced by greater inclusion into mainstream society, a drop in infant mortality rate, advances in medical care, and positive changes related to overall health [3,4,5,6,7]. According to the literature [1,2], alterations such as atrioventricular septal defects in the heart, leukemia, and early-onset Alzheimer’s disease (AD), among others, are common in this population.

Although diagnosis of dementia in DS individuals occurs, in general, after the age of 60 [8], cognitive decline can already be observed after age 40 [9], including alterations in memory, attention, executive functioning, motor planning, and coordination [10,11].

A multidisciplinary study [12] showed the severity of behavioral changes throughout the life of people with DS in relation to dementia. Although the study demonstrated a scenario associated with disorders such as anxiety, sleep disorders, and depressive symptoms, there was still a question regarding the condition of dementia and its diagnosis.

AD is characterized by extracellular senile plaques and intracellular neurofibrillary tangles (NFTs), which lead to loss of neurons and synapses. The increased risk of developing AD in people with DS is related to the presence of an extra copy of the amyloid precursor protein (APP) gene on chromosome 21 [8], leading to overproduction of amyloid beta peptide (Aβ) [13]. Aβ peptides are produced from proteolytic cleavages of APP and this overproduction results in an increase in plaque formation [14,15,16,17]. Inflammation, oxidative stress, and gliosis are also pathological mechanisms described in the literature as contributing to the neurodegeneration process [8,15,16,17].

People with DS present early amyloid deposition, associated with AD in the temporal neocortex, when compared with non-DS individuals [8,16,17,18]. Some epigenetic mechanisms, such as DNA methylation and nuclear reorganization, could also be associated with the pathogenesis of AD in DS [13,19,20,21,22,23].

There is a lack of potential treatments for dementia in people with DS, mainly when involving randomized controlled trials. The present review aims to synthesize the different pharmacological therapeutic approaches published in the last 20 years, in order to improve the set of symptoms related to dementia in people with DS.

## 2. Materials and Methods

Randomized controlled trials were selected that involved adult participants with DS, with any type of pharmacological treatment for improving the symptoms related to dementia. The searches included the period from 2002 to 2022 (the last twenty years).

The studies were considered for this review according to the inclusion of adult participants with diagnosed DS through validated instruments and application of a pharmacological treatment compared to a placebo group to improve the set of symptoms associated with dementia. Studies were excluded according to the following items: articles that did not qualify as randomized controlled trials, studies involving young people with DS and no sign of dementia, and animal model studies.

Electronic searches were performed in the following databases: MEDLINE, EMBASE, the Cochrane Library (CENTRAL), and Web of Science. Only studies in Portuguese and English were selected. The searches were performed until April 2022. The search strategy was developed using the following keyword combinations (Mesh): (Down syndrome) OR (trisomy 21) AND (dementia); (Down syndrome) OR (trisomy 21) AND (Alzheimer disease); (Down syndrome) AND (dementia) AND (treatment); (Down syndrome) AND (Alzheimer disease) AND (treatment). When necessary, the search strategy was adapted to each database.

We used the search strategy according to the PICOS [24] (population (P), intervention (I), control group (C), outcome (O), and study design (S)) method to compare different studies: P—down syndrome/I—pharmacological therapy/C—down syndrome with a placebo drug/O- cognitive improvement/S—randomized controlled trials.

The following data were extracted: publication, study design, characteristics of participants (age, sample size), and interventions performed (drug, dose, duration of treatment), outcome, and main conclusions (Table 1).

The quality review was performed according to the Cochrane Collaboration tool (Table 2) for assessing the risk of bias in randomized controlled trials. Each item of this tool is explained below [25]:

“Random sequence generation” = Described the method used to generate the allocation sequence with details.

“Allocation concealment” = Described the method used to conceal the allocation sequence, with details. The objective is to determine whether the intervention allocations could have been foreseen prior to or during enrollment.

“Blinding of participants and personnel” = In this question, all measures used are described, which intervention a participant received, and other important points.

“Blinding of outcome assessment” = In this question, all measures related to blinding outcome assessors from knowledge of which intervention a participant received are described.

“Selective reporting” = This item demonstrates how the selective outcome was examined by the authors and what was found.

“Incomplete outcome data” = Cites any attrition and exclusions from the analysis and the numbers in each intervention group.

“Anything else, ideally prespecified” = Any important concerns about bias are cited.

The evaluation of this tool is based on “Low risk of bias”, “Unclear risk of bias”, and “High risk of bias” described below [26]:

- “Low risk of bias” = The trial is judged to be at a low risk of bias for all domains for this result.
- “Unclear” = The trial is judged to raise some concerns in at least one domain for this result, but not to be at a high risk of bias for any domain.
- “High risk of bias” = The trial is judged to be at a high risk of bias in at least one domain for this result.

Studies performed between 2002 and 2022 were selected. Due to the small number of randomized controlled trials and the great heterogeneity among them, the studies were analyzed qualitatively, without meta-analysis. The PRISMA recommendations were followed to perform this systematic review [24].

**Table 1:** Characteristics of the included studies.

Publication	Study Design	Participants		Pharmacological Treatment			Outcome	Main Conclusions
		Age (Mean ± SD)	Sample Size (Participants with DS)	Drug	Dose	Duration		
Prasher et al. (2002) [27]	A randomized, double-blind, placebo-controlled study	53 ± 8.03	30	Donepezil	5 mg per day during the first four weeks and then 10 mg per day thereafter	24 weeks	↓ *NPI* * (Improvement in the treated group although less significant than placebo group)	There is possible efficacy in the treatment of symptoms of mild to moderate AD with the use of donepezil in people with DS
Lott et a. (2011) [3]	A randomized, double-blind, placebo-controlled study	50 ± 4.88	53	Antioxidant supplementation	900 IU Alpha tocopherol, 200 mg ascorbic acid followed by 600 mg alpha—lipoic acid. All participants received an associated acetylcholinesterase inhibitor	2 years	*DMR* *, *SIB* *, *DMR SOC* *, *BADLS* *, and *BPT* * (No significant differences between groups) ↓ *VABS* * *motor skills* (Significant difference in the treated group in 2-year of treatment)	Antioxidant supplementation is safe, however, ineffective for the treatment of dementia in people with DS and dementia of the Alzheimer’s type
Kondoh et al. (2011) [28]	A randomized, double-blind, placebo-controlled clinical trial	45 **	21	Donepezil	3 mg once daily throughout the trial	24 weeks	↑ *ICF* * (improvement only in the treated group)	Donepezil can help improve general functioning and severe cognitive impairment effectively and safely in people with DS
Hanney et al. (2012) [29]	A randomized, double-blind, placebo-controlled trial	51 ± 7.3	173	Memantine	The dose was escalated over 8 weeks from 5 mg per day to the optimal therapeutic dose of 10 mg per day with fixed titration	52 weeks	*DAMES* *, *ABS* * Non-significant differences between the groups	Memantine is not an effective treatment for cognitive impairment and dementia in people older than 40 years with DS
Sano et al. (2016) [30]	A randomized, double-blind, controlled clinical trial	54 ± 4.75	337	Vitamin E	1000 IU orally twice daily	3 years	*BPT* * and memory tests (both verbal and visual), Vocabulary Test, Orientation Test, The Behavior & Function Questionnaire, and *CGI-C* * No differences between the groups	Vitamin E did not slow the progression of cognitive deterioration in DS
Rosenbloom et al. (2020) [4]	A single-center, single-dose, randomized, double-blind, placebo-controlled, crossover pilot study	42 ± 1.7	12	Intranasal insulin	A total of 0.20 mM of glulisine or placebo was administered using the POD® device to deliver 0.10 mL of agent in each nostril for a total of 20 IU	8 weeks	↑ RBMT * There was significant improvement in memory retention in the glulisine treated group and in immediate recall in the placebo group FOME * No significant impact in the groups	There was no significant impact of intranasal glulisine on learning, immediate recall, delayed recall, memory retention, recognition memory, and retention estimate

Abbreviations * *DMR* Dementia Scale for Intellectually Disabled Persons. *SIB* Severe Impairment Battery. *NPI* Neuropsychiatric Inventory. *ABS* Adaptive Behavior Scale. *DMR SOS* The Sum of Social Scores on the Dementia Scale for Mentally Retarded Persons. *VABS* The Vineland Adaptive Behavior Scales. *BADLS* The Bristol Activities of Daily Living Scale. *BPT* Brief Praxis Test. *ICF* International Classification of Functioning, Disability and Health. *DAMES* Down syndrome attention, memory and executive function scale. *CGI-C* The Clinical Global Impression of Change. *RBMT* Rivermead Behavioral Memory Test. *FOME* Fuld Object Memory Evaluation. ** (SD) was not cited in the study. ↑ Increase/↓ Decrease.

**Table 2:** Risk of bias.

	Random Sequence Generation	Allocation Concealment	Blinding of Participants and Personnel	Blinding of Outcome Assessment	Incomplete Outcome Data	Selective Reporting	Anything Else, Ideally Prespecified
Prasher et al. (2002) [27]	Low	Low	Low	Low	Low	Low	Low
Lott et al. (2011) [3]	Low	Low	Low	Low	Low	Low	Low
Kondoh et al. (2011) [28]	Low	Low	Low	Low	Low	Low	Low
Hanney et al. (2012) [29]	Low	Low	Low	Low	Low	Low	Low
Sano et al. (2016) [30]	Low	Low	Low	Unclear	Low	Unclear	Low
Rosenbloom et al. (2020) [4]	Low	Low	Low	Unclear	Low	Low	Low

## 3. Results

**Figure 1:**
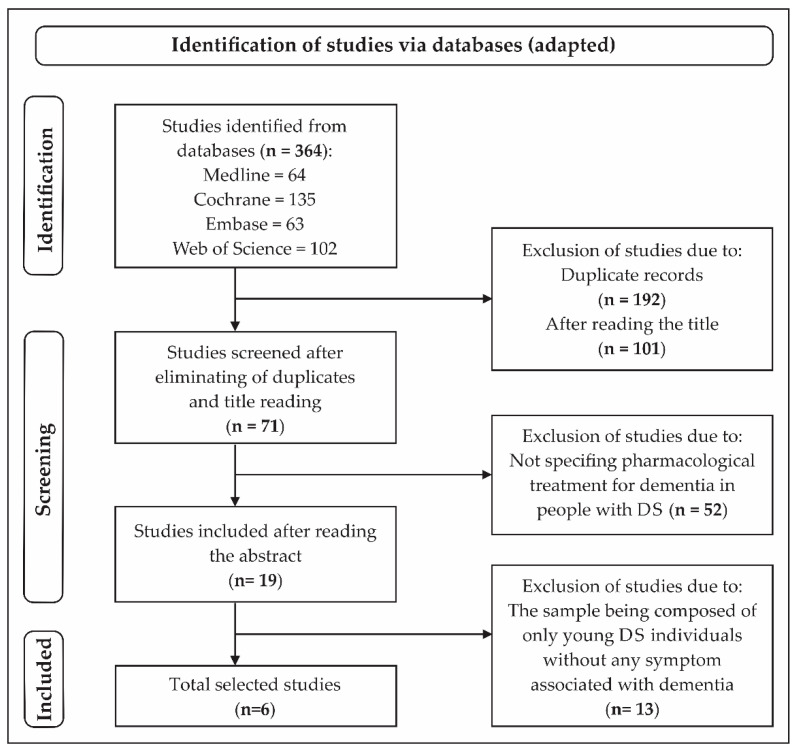
Flowchart of the search process of the selected studies [28].

Initially, 364 studies were selected. After eliminating duplicate papers and analyzing the titles, 71 studies were included. Of these, 52 were eliminated because they did not specify any pharmacological treatment for dementia in people with DS. After reading the abstract, 19 studies were selected, and after full reading, 13 were eliminated because the individuals with DS were young, without any symptom associated with dementia. Thus, in the final phase, six studies were included (Figure 1).

The average age of the participants with DS included in this review was 50 years [3,4,27,28,29,30]. In relation to methodological quality, the risk of bias was low in all the articles evaluated, excepted for “unclear” in some analyzed criteria in two of them [4,30].

In this review, important points about the effectiveness of different pharmacological treatments for adults with DS, focusing on AD signs, were summarized; among them were the use of supplemental antioxidant therapies [3], donepezil [27,28], memantine [29], vitamin E [30], and intranasal insulin [4]. The effects of each treatment are reported in Table 1. Donepezil was the only drug with a possible effect on the treatment of cognitive signs of dementia in adults with DS [27,28].

In the studies with memantine [29] and donepezil [27,28], more adverse effects were reported than with the other drugs presented in this review. Diarrhea, nausea, insomnia, fatigue [27], soft stool, skin rash [28], and adverse events associated with neurological, respiratory, and cardiovascular alterations [29] were identified.

On the whole, randomized clinical studies that evaluate new pharmacological therapies to improve the set of symptoms associated with dementia are scarce in adults with DS. This lack represents an important public health problem, as an absence of effective treatments has a direct impact on the quality of life of these individuals, with important repercussions from an economic point of view and on the rates of mortality and morbidity [31,32].

## 4. Discussion

In this review, we included only randomized controlled clinical trials that reflected new perspectives on the use of pharmacological therapies for adults with DS with symptoms associated with dementia. A limitation regarding this type of study is reported in the literature, mainly due to the difficulty of recruitment, as well as the complexity of diagnosis in DS population [33,34].

People with DS have a higher risk of developing AD than the population without the syndrome and studies based on effective treatments for this pathology are important [34]. Vitamin E, for example, has important neuroprotective, anti-inflammatory, and hypocholesterolemic effects in brain health. Besides this, it has great antioxidant potential against peroxyl radicals and can enhance the immune response in older people [35,36,37,38]. Only one study [30] in this systematic review evaluated the effect of vitamin E. The authors observed a lack of contribution to slowing the progression of cognitive deterioration and dementia in people with DS [30], which corroborates the finding of Petersen et al. (2005) [39], who also evaluated the efficacy of vitamin E associated with another drug, donepezil, in individuals with amnestic mild cognitive impairment but not DS. In that study [39], vitamin E had no significant effect in relation to the development of AD, but the analysis for donepezil, however, demonstrated reduced progression of AD in the first 12 months of the trial.

Memantine is a drug used for improving symptomatic AD, acting in the maintenance of cognitive function in the general population [40,41]. This drug, when analyzed in the population with DS, did not present similar effects [29]. One hypothesis is that AD in DS is related to lifelong amyloid overproduction, similar to some rare familial forms of AD, and different from late-onset sporadic AD that presents abnormalities in amyloid processing and clearance [29].

Some studies associated with the use of donepezil and memantine in people with DS were not included in this systematic review because they did not meet the inclusion criteria. For example, Lott et al. (2002) [42], showed that the use of donepezil caused significant improvement in cognitive functioning in people with DS during a period of 3 to 5 months (average age of 52.3 in the donepezil group), but the study was not included in this review because it is a non-randomized controlled trial. Two randomized clinical trials [43,44] associated with the use of memantine in young people with DS were in line with the study with older participants reported in this systematic review [29], which also demonstrated no cognitive improvement. These studies were not included because the participants were young, with no symptoms related to dementia.

Many factors could be associated with the heterogenous results found in the evaluated studies. The dosage and duration of the medication used and the gravity of the set of symptoms related to dementia could be correlated with the differences observed [34]. Rosenbloom et al. (2020) [4] reported the importance of analyzing factors such as the daily dose of the drug and the longitudinal duration of treatment for a better response of the agent in regulating pathological changes in dementia cases. In their study, the authors detected no significant effect of the rapid-acting intranasal insulin on delayed recall and memory recognition between individuals with DS and a placebo group. In AD, there is progressive glucose hypometabolism parallel to cognitive impairment, and insulin plays a major neuroprotective role, countering apoptosis, beta-amyloid toxicity, and oxidative stress [45].

Studies with other potential drugs for the treatment of dementia are reported in the literature [46,47,48,49,50]. Galantamine and rivastigmine appear to have some clinical effect in the treatment of mild to moderate AD [46,47]; however, a lack of information is reported regarding the use of these drugs for people with intellectual disability [48]. Cochrane reviews [49,50] also show no significant evidence of these drugs for the treatment of dementia in people with DS. During the current review, no randomized clinical trials were found that contemplated the use of galantamine and rivastigmine in the population with DS, according to the proposed inclusion criteria.

Studies related to novel cellular protective mechanisms associated with the neuropathology of AD in DS and ongoing clinical trials that portray new perspectives for its treatment must be explored [51,52,53]. The literature reports evaluations of the dual-specificity tyrosine phosphorylation-regulated kinase 1A (DYRK1A), a gene mapped on chromosome 21 that contributes to the hyperphosphorylation of tau and the formation of hyperphosphorylated tau aggregates. Furthermore, DYRK1A phosphorylates several neurodegenerative diseases associated proteins, such as APP and α-synuclein, and has been associated with intellectual disability and targeted to improve cognitive performance in subjects with DS (without AD) and other intellectual disabilities. New possibilities related to cell protection are demonstrated, when the presence of other binding proteins, such as protein phosphatase magnesium-dependent 1B (PPM1B), reduces the toxic formation of phospho-tau protein via DYRK1A modulation [51,54].

Furthermore, a randomized pilot study [52], still in the recruitment phase, is being conducted to evaluate the efficacy of therapy with gonadotrophin-releasing hormone (GnRH), a decapeptide secreted by hypothalamic neurons, on the cognition of people with DS. The presence of this and other related papers suggests the need for further exploration of points not yet analyzed, mainly associated with the early deposition of β-amyloid plaques and development of dementia-related AD [34].

This systematic review did not address the preventive treatment of dementia in people with DS. In all included studies, the selected individuals with DS were adult with some symptoms associated with dementia. The term “preventive treatment” is related to a set of actions, involving, on the whole, both pharmacological and non-pharmacological therapies. It is important to elucidate whether individuals with DS of any age and with any symptom of dementia (even an initial behavior alteration) would benefit from studies focused on pharmacological and/or non-pharmacological therapies applied in larger samples sizes over a longer period.

## 5. Conclusions

The treatment of dementia in DS individuals is a field that still requires more studies, mainly due to the difficulty of early diagnosis and follow-up of its evolution. In general, the results of this review demonstrated that the use of pharmacological therapies for symptoms related to dementia did not cause relevant effects on different functions in the population with DS. For example, aspects related to memory and general functionality of these individuals did not show improved performance after the application of a pharmacological therapy in almost any of the studies analyzed. However, more studies are necessary to draw conclusions about possible targets for treatment, focusing on AD in the DS population, and the limited number of studies included in this review shows the eminent necessity for further investigations on this subject. On the whole, the drugs were well tolerated by individuals with DS; however, the majority did not demonstrate adequate efficacy. The use of antioxidant supplementation, memantine, vitamin E, and intranasal insulin did not have relevant effects for the treatment of dementia in people with DS.

Positive changes related to some general functions in people with DS were observed in two randomized clinical trials, related to the use of donepezil. Treatments targeting the tau and amyloid-beta mechanisms for people with DS should be further explored in the literature, as well as quality of life aspects and expansion of available biomarkers.

## References

[1] Sinet P.M., Theopile D., Rahmani Z., Chettouch Z., Blovin J.L., Prier M., Noel B., Delabar J.M. (1994). Mapping of Down syndrome phenotype on chromosome 21 at themolecular level. Biomed. Pharmacother..

[2] Asim A., Kumar A., Muthuswamy S., Jain S., Agarwal S. (2015). Down syndrome: An insight of the disease. J. Biomed.Sci..

[3] Lott I.T., Doran E., Nguyen V.Q., Tournay A., Head E., Gillen D.L. (2011). Down syndrome and dementia: A randomized, controlled trial of antioxidant supplementation. Am. J. Med. Genet. Part A.

[4] Rosenbloom M., Barclay T., Johnsen J., Erickson L., Svitak A., Pyle M., Frey W., Hanson L.R. (2020). Double-Blind Placebo-Controlled Pilot Investigation of the Safety of a Single Dose of Rapid-Acting Intranasal Insulin in Down Syndrome. Drugs RD.

[5] Parizot E., Dard R., Janel N., Vialard F. (2019). Down syndrome and infertility: What support should we provide?. J. Assist. Reprod. Genet..

[6] Bittles A.H., Bower C., Hussain R., Glasson E.J. (2007). The four ages of Down syndrome. Eur. J. Public Health.

[7] Rafii M.S., Santoro S.L. (2019). Prevalence and Severity of Alzheimer Disease in Individuals with Down Syndrome. JAMA Neurol..

[8] Rumble B. (1989). Retallak, R. Amyloid A4 and its precursor in Down’s syndrome and Alzheimer’s disease. N. Engl. J..

[9] Strydom A., Coppus A., Blesa R., Danek A., Fortea J., Hardy J., Levin J., Nuebling G., Rebillat A.S., Ritchie C. (2018). Alzheimer’s disease in Down syndrome: An overlooked population for prevention trials. Alzheimer’s Dement. Transl. Res. Clin. Interv..

[10] Cody K.A., Piro-Gambetti B., Zammit M.D., Christian B.T., Handen B.L., Klunk W.E., Zaman S., Johnson S.C., Plante D.T., Hartley S.L. (2020). Association of sleep with cognition and beta amyloid accumulation in adults with Down syndrome. Neurobiol. Aging.

[11] Startin C.M., Hamburg S., Hithersay R., Al-Janabi T., Mok K.Y., Hardy J., Strydom A., Fisher E., Nizetic D., Tybulewicz V. (2019). Cognitive markers of preclinical and prodromal Alzheimer’s disease in Down syndrome. Alzheimers Dement..

[12] Dekker A.D., Sacco S., Carfi A., Benejam B., Vermeiren Y., Beugelsdijk G., Schippers M., Hassefras L., Eleveld J., Grefelman S. (2018). The Behavioral and Psychological Symptoms of Dementia in Down Syndrome (BPSD-DS) Scale: Comprehensive Assessment of Psychopathology in Down Syndrome. J. Alzheimer’s Dis..

[13] Wiseman K., Al-Janabi T., Hardy J., Karmiloff-Smith A., Nizetic D., Tybulewicz V.L., Fisher E., Strydom A. (2015). A genetic cause of Alzheimer disease: Mechanistic insights from Down syndrome. Nat. Rev. Neurosci..

[14] Wiseman F.K., Pulford L.J., Barkus C., Liao F., Portelius E., Webb R., Chávez-Gutiérrez L., Cleverley K., Noy S., Sheppard O. (2018). Trisomy of human chromosome 21 enhances amyloid-beta deposition independently of an extra copy of APP. Brain.

[15] Cacace R., Sleegers K., Van Broeckhoven C. (2016). Molecular genetics of early-onset Alzheimer’s disease revisited. Alzheimers Dement..

[16] Wisniewski K.E., Wisniewski H.M., Wen G.Y. (1985). Occurrence of neuropathological changes and dementia of Alzheimer’s disease in Down’s syndrome. Ann. Neurol..

[17] Davidson Y.S., Robinson A., Prasher V.P., Mann D.M.A. (2018). The age of onset and evolution of Braak tangle stage and Thal amyloid pathology of Alzheimer’s disease in individuals with Down syndrome. Acta Neuropathol. Commun..

[18] Mann D.M. (1988). The pathological association between Down syndrome and Alzheimer disease. Mech. Ageing Dev..

[19] Griffiths-Jones S. (2004). The microRNA Registry. Nucleic Acids Res..

[20] Rachidi M., Lopes C. (2010). Molecular and cellular mechanisms elucidating neurocognitive basis of functional impairments associated with intellectual disability in Down syndrome. Am. J. Intellect. Dev. Disabil..

[21] Della Ragione F., Gagliardi M., D’Esposito M., Matarazzo M.R. (2014). Non-coding RNAs in chromatin disease involving neurological defects. Front. Cell Neurosci..

[22] Hamlett E.D., Ledreux A., Potter H., Chial H.J., Patterson D., Espinosa J.M., Bettcher B.M., Granholm A.C. (2018). Exosomal biomarkers in Down syndrome and Alzheimer’s disease. Free Radic. Biol. Med..

[23] Matsuoka Y., Andrews H.F., Becker A.G., Gray A.J., Mehta P.D., Sano M.C., Dalton A.J., Aisen P.S. (2009). The relationship of plasma Abeta levels to dementia in aging individuals with Down syndrome. Alzheimer Dis. Assoc. Disord..

[24] Liberati A., Altman D.G., Tetzlaff J., Mulrow C., Gøtzsche P.C., Ioannidis J.P.A., Clarke M., Devereaux P.J., Kleijnen J., Moher D. (2009). The PRISMA statement for reporting systematic reviews and meta-analyses of studies that evaluate healthcare interventions: Explanation and elaboration. BMJ.

[25] Higgins J.P.T., Altman D.G., Gøtzsche P.C., Jüni P., Moher D., Oxman A.D., Savović J., Schulz K.F., Weeks L., Sterne J.A. (2011). The Cochrane Collaboration’s tool for assessing risk of bias in randomised trials. BMJ.

[26] Higgins J.P.T., Thomas J., Chandler J., Cumpston M., Li T., Page M.J., Welch V.A. (2021). Cochrane Handbook for Systematic Reviews of Interventions.

[27] Prasher V.P., Huxley A., Haque M.S., Down Syndrome Ageing Study Group (2002). A 24-week, double-blind, place-controlled trial of donepezil in patients with Down syndrome and Alzheimer’s disease—Pilot study. Int. J. Geriatr. Psychiatry.

[28] Kondoh T., Kanno A., Itoh H., Nakashima M., Honda R., Kojima M., Noguchi M., Nakane H., Nozaki H., Sasaki H. (2011). Donepezil significantly improves abilities in daily lives of female down syndrome patients with severe cognitive impairment: A 24-week randomized, double-blind, placebo-controlled trial. Int. J. Psychiatry Med..

[29] Hanney M., Prasher V., Williams N., Jones E.L., Aarsland D., Corbett A., Lawrence D., Yu L.M., Tyrer S., Francis P.T. (2012). Memantine for dementia in adults older than 40 years with Down’s syndrome (MEADOWS): A randomised, double-blind, placebo-controlled trial. Lancet.

[30] Sano M., Aisen P.S., Andrews H.F., Tsai W., Lai F., Dalton A.J. (2016). Vitamin E in Aging Persons with Down Syndrome. Neurology.

[31] Daly B., Watt R., Batchelor P., Treasure E. (2002). Essential Dental Public Health.

[32] Leavell H.R., Leavell H.R., Clark E.G. (1976). Planejamento para a Saúde Comunitária. Medicina Preventiva.

[33] Ellis J.M., Tan H.K., Gilbert R.E., Muller D.P.R., Henley W., Moy R., Pumphrey R., Ani C., Davies S., Edwards V. (2008). Supplementation with antioxidants and folinic acid for children with Down’s syndrome: Randomised controlled trial. BMJ.

[34] Castro P., Zaman S., Holland A. (2017). Alzheimer’s disease in people with Down’s syndrome: The prospects for and the challenges of developing preventative treatments. J. Neurol..

[35] Brigelius-Flohe R., Traber M.G. (1999). Vitamin E: Function and metabolism. FASEB J..

[36] Reiter E., Jiang Q., Christen S. (2007). Anti-inflammatory properties ofα- andγ-tocopherol. Mol. Asp. Med..

[37] Jiang Q. (2014). Natural forms of vitamin E: Metabolism, antioxidant, and anti-inflammatory activities and theirrole in disease prevention and therapy. Free Radic. Biol. Med..

[38] Xu L., Davis T.A., Porter N.A. (2009). Rate constants for peroxidation of polyunsaturated fatty acids and sterols insolution and in liposomes. J. Am. Chem. Soc..

[39] Petersen R.C., Thomas R.G., Grundman M., Bennett D., Doody R., Ferris S., Galasko D., Jin S., Kaye J., Levey A. (2005). Vitamin E and donepezil for the treatment of mild cognitive impairment. N. Engl. J. Med..

[40] Lu S., Nasrallah H.A. (2018). The use of memantine in neuropsychiatric disorders: An overview. Ann. Clin. Psychiatry.

[41] Kishi T., Matsunaga S., Oya K., Nomura I., Ikuta T., Iwata N. (2017). Memantine for Alzheimer’s Disease: An Updated Systematic Review and Meta-analysis. J. Alzheimers Dis..

[42] Lott I.T., Osann K., Doran E., Nelson L. (2002). Down syndrome and Alzheimer’s disease: Response to donepezil. Arch. Neurol..

[43] Boada R., Hutaff-Lee C., Schrader A., Weitzenkamp D., Benke T.A., Goldson E.J., Costa A.C. (2012). Antagonism of NMDA receptors as a potential treatment for Down syndrome: A pilot randomized controlled trial. Transl Psychiatry.

[44] Costa A.C.S., Brandão A.C., Boada R., Barrionuevo V.L., Taylor H.G., Roth E., Stasko M.R., Johnson M.W., Assir F.F., Roberto M.P. (2022). Safety, efficacy, and tolerability of memantine for cognitive and adaptive outcome measures in adolescents and young adults with Down syndrome: A randomised, double-blind, placebo-controlled phase 2 trial. Lancet Neurol..

[45] Berlanga-Acosta J., Guillén-Nieto G., Rodríguez-Rodríguez N., Bringas-Vega M.L., García-del-Barco-Herrera D., Berlanga-Saez J.O., García-Ojalvo A., Valdés-Sosa M.J., Valdés-Sosa P.A. (2020). Insulin Resistance at the Crossroad of Alzheimer Disease Pathology: A Review. Front. Endocrinol..

[46] National Institute for Health and Clinical Excellence (2007). Donepezil, Galantamine, Riv-Astigmine (Review) and Memantine for the Treatment of Alzheimer’s Disease (Amended).

[47] Clegg A., Bryant J., Nicholson T., McIntyre L., De Broe S., Gerard K., Waugh N. (2002). Clinical and cost-effectiveness of donepezil, rivastigmine, and galantamine for Alzheimer’s disease. A systematic review. Int. J. Technol. Assess. Health Care.

[48] Prasher V.P. (2004). Review of donepezil, rivastigmine, galantamine and memantine for the treatment of dementia in Alzheimer’s disease in adults with Down syndrome: Implications for the intellectual disability population. Int. J. Geriatr. Pshychiatry.

[49] Mohan M., Bennett C., Carpenter P.K. (2009). Galantamine for dementia in people with Down syndrome. Cochrane Database Syst. Rev..

[50] Mohan M., Bennett C., Carpenter P.K. (2009). Rivastigmine for dementia in people with Down syndrome. Cochrane Database Syst. Rev..

[51] Lee Y.H., Im E., Hyun M., Park J., Chung K.C. (2021). Protein phosphatase PPM1B inhibits DYRK1A kinase through dephosphorylation of pS258 and reduces toxic tau aggregation. J. Biol. Chem..

[52] Nelly Pitteloud, Centre Hospitalier Universitaire Vaudois Clinical Trials Registry [Internet]: Nelly Pitteloud: Clinical Trials Centre, Universitaire Vaudois (Suiça) (2020). Identifier NCT04390646 History of Changes. GnRH Therapy on Cognition in Down Syndrome. NCT04390646.

[53] Rondal J.A. (2020). Down syndrome: A curative prospect?. AIMS Neurosci..

[54] Park J., Song W.J., Chung K.C. (2009). Function and regulation of Dyrk1A: Towards understanding Down syndrome. Cell Mol. Life Sci..

